# Multiplatform Metabolomics for the Design and Characterization of a Mediterranean Plant-Based Lyophilized Powder from Agro-Industrial By-Products

**DOI:** 10.3390/foods15030565

**Published:** 2026-02-05

**Authors:** Rosa Toledo-Gil, Pasquale Crupi, Jose Enrique Yuste-Jiménez, Fernando Vallejo

**Affiliations:** 1 Metabolomics Platform, Centro de Edafología y Biología Aplicada del Segura (CEBAS-CSIC), Campus Universitario de Espinardo, 30100 Murcia, Spain; rtoledo@cebas.csic.es (R.T.-G.); jyuste@cebas.csic.es (J.E.Y.-J.); 2 Department of Agricultural, Food and Forestry Sciences, University of Palermo, 90128 Palermo, Italy; pasquale.crupi@unipa.it

**Keywords:** plant-based by-products, BIOMEDER, phytochemicals, nutrients, VOCs, UPLC-QTOF-MS, UHPLC-QTRAP-MS, ^1^H-NMR spectroscopy, SPE-GC-MS

## Abstract

Agri-food industries generate substantial quantities of side streams such as peels, pods, seeds, and leaves. Traditionally regarded as waste, these by-products are now recognized as rich sources of bioactive compounds—often at higher concentrations than those found in edible plant parts. Their recovery reduces environmental impact and enables the development of sustainable ingredients for food and health-related applications, in line with circular economy principles. This study presents the design and metabolomic characterization of a novel lyophilized powder derived from Mediterranean and locally cultivated plant-based by-products (named BIOMEDER), including orange, lemon, olive leaves, carob pods, shiitake mushroom, and salicornia. A multiplatform metabolomics approach was applied, combining high-resolution UPLC-QTOF-MS, UHPLC-QTRAP-MS, SPME-GC-MS, and ^1^H-NMR spectroscopy to comprehensively profile phytochemicals, nutrients, and volatile organic compounds (VOCs). The powder was found to be rich in flavonoids (e.g., luteolin-7-O-glucoside, hesperidin, eriocitrin), phenolic acids, amino acids (e.g., proline, GABA), organic acids (e.g., malic and citric acid), and over 40 VOCs associated with antioxidant and sensory functions. Notably, high concentrations of these compounds suggest potential health-promoting properties. These findings might support the formulation of a potential functional plant-based supplement and reinforce the value of integrating diverse agro-industrial by-products into sustainable, health-oriented food solutions.

## 1. Introduction

The increasing demand for sustainable and functional ingredients has driven both the scientific community and the food industry to explore the valorization of fruit and vegetable by-products. These plant-based materials, often discarded during industrial processing, are rich in bioactive compounds—particularly phenolic metabolites—which have been associated with antioxidant, anti-inflammatory, and antimicrobial activities beneficial to human health [1,2]. Transforming these residues into functional ingredients not only enhances the nutritional value of food products but also aligns with circular economy strategies aimed at minimizing environmental impact [3,4]. Among the most widely generated by-products are those from citrus fruits (*Citrus* spp.), with global production exceeding 90 million tons annually. Nearly one-third of this yield is processed into juice, resulting in massive volumes of peel and pulp residues [5]. These residues are rich in flavanones such as hesperidin, eriocitrin, and narirutin (20–50 mg/g d.w), as well as flavones, flavonols, and polymethoxylated flavones—compounds linked to cardioprotective, anti-obesity, and antiviral effects [6,7,8,9]. Similarly, olive leaves are notable for their high concentrations of oleuropein, hydroxytyrosol, and verbascoside (8–15 mg/g d.w.)—potent antioxidants with applications in food supplements and nutraceuticals [10]. Carob pods (*Ceratonia siliqua* L.), often underutilized, contain significant levels of gallic acid (3–6 mg/g), catechins, and quercetin derivatives, contributing to their antioxidant and antimicrobial potential. Carob is also being promoted as a caffeine-free alternative to cocoa in functional formulations [11,12]. Mushroom cultivation, especially from species like shiitake (*Lentinula edodes*), generates bioresidues rich in polysaccharides and phenolic acids (8–11 mg/g) with immunomodulatory and antioxidant properties [13,14]. Furthermore, halophytic plants such as *Salicornia europaea* have recently gained attention due to their unique phenolic profile, mineral content, and traditional use in managing metabolic and inflammatory conditions [15]. Collectively, these by-products not only offer a wide phytochemical diversity but also represent a considerable portion of food processing waste, where citrus processing generates around 15 million tons of waste annually in the Mediterranean region [5]. This individual nutritional and functional richness supports their strategic combination into a single powder that may be functional.

Despite the promising individual profiles of these by-products, their combination remains underexplored. Integrating residues from orange, lemon, olive leaves, carob pods, mushroom, and salicornia might result in enhanced bioactivity, offering enhanced health benefits compared to single-source powders. Unlocking this potential requires advanced analytical tools capable of comprehensive metabolite profiling. To this end, state-of-the-art metabolomics platforms—such as high-resolution UPLC-QTOF-MS, high-sensitivity UHPLC-QTRAP-MS, ^1^H-NMR, and SPME-GC-MS—are essential for accurate qualitative and quantitative characterization of complex phytochemical and nutrient profiles. Their combined application ensures accurate characterization of the chemical diversity in novel supplements, which is essential for reproducibility, safety assessment, and regulatory acceptance in functional foods [16,17].

The objectives of this study were threefold:

(i) To design a novel lyophilized powder from six Mediterranean plant-based by-products (orange, lemon, olive leaves, carob pods, shiitake mushroom, and salicornia) that might be multifunctional;
(ii) To perform a detailed metabolomic profiling using complementary analytical platforms;
(iii) To evaluate the diversity of phytochemicals, nutrients, and volatile organic compounds (VOCs), as well as their potential functional relevance in the context of sustainable food systems.

## 2. Materials and Methods

### 2.1. Reagents

Analytical HPLC-MS-grade chemicals, including water with 0.1% formic acid, acetonitrile (ACN), dimethyl sulfoxide (DMSO), and methanol (MeOH), were obtained from J.T. Baker (Deventer, The Netherlands). The following 38 pure standards were purchased from Extrasynthese (Genay, France): Catechin, Gallocatechin, Procyanidin dimer B1, Theaflavin, Didymin, Eriocitrin, Eriodictyol, Hesperetin, Hesperidin, Naringenin, Neoeriocitrin, Neohesperidin, Poncirin, Apigenin, 6-Hydroxyluteolin, Chrysin, Luteolin, Nobiletin, Sinensetin, Tangeretin, Isorhamnetin, Kaempferol, Myricetin, Quercetin, Rhamnetin, Daidzein, Daidzin, 2,3-Dihydroxybenzoic acid, 2-Hydroxybenzoic acid, Benzoic acid, Ellagic acid, Gallic acid, Vanillic acid, Caffeic acid, Ferulic acid, p-Coumaric acid, Rosmarinic acid and Sinapic acid.

### 2.2. Designing a New Lyophilized Powder (BIOMEDER)

Four Mediterranean plant-based by-products from Spain, orange (*Citrus sinensis* L.), lemon (*Citrus limon* L.), shiitake mushroom (*Lentinula edodes* L.) and salicornia (*Salicornia europaea* L.), and two from Italy, olive leaves (*Olea europaea* L.) and carob pods (*Ceratonia siliqua* L.), were frozen at −80 °C and lyophilized using a [Telstar/lyoquest-55] freeze-dryer under −85 °C and 0.1 mbar for 3 days and ground with a mixer grinder to design a homogenized powder complemented in phytochemicals, nutrients and organic volatile compounds (VOCs). Spanish by-products were provided by a local company (Agrosingularity, Murcia, Spain); meanwhile, Italian by-products were provided by Department of Agricultural, Food and Forestry Sciences, University of Palermo. This new designed homogenized powder was named BIOMEDER (BIOactive MEditerranean powDER) and it is patent-pending.

The formulation of BIOMEDER was defined using an evidence-based approach. The relative proportions of each by-product are expressed as weight/weight (*w*/*w*) percentages on a dry-weight basis per capsule (300 mg). These proportions were established (i) to obtain a mixture containing the widest and diverse range of bioactive, nutritional and volatile aromatic compounds possible combining major by-products (citrus and olive leaves) and emerging ones (carob pods, salicornia and shiitake mushroom), and (ii) in order to follow the most objective and realistic criteria, the design strategy was to give prominence to both citrus and olive by-products, as they are one of the main sources of by-products per ton/year (≈50–70% of total) [5,10] and also constituted a rich source of phenolic compounds and VOCs [10]. For these two reasons, they represented a final 70% of the powder. The remaining 30% was distributed equally among three other emerging by-products (shiitake, salicornia and carob), as their availability is already very scarce compared to the others. Thus, the final mixed composition expressed in dry weight per capsule of the lyophilized powder was the following: olive leaves (90 mg—30%), orange (60 mg—20%), lemon (60 mg—20%), carob pods (30 mg—10%), salicornia (30 mg—10%) and shiitake mushroom (30 mg—10%). In order to obtain a homogenized powder, the mixed was vortexed (Cole Parmer, Vernon Hills, IL, USA) twice for 5 min. A final weight of 300 mg was selected for filling sized—1 hard gelatin capsules supplied by Blendhub S.L. (Murcia, Spain), following the European Union’s good manufacturing practices. Each capsule contained more than 150 mg of bioactive phytochemical compounds. No factorial or response surface experimental design was applied to optimize the formulation. Therefore, the composition should be interpreted as a knowledge-driven formulation prototype, intended for comprehensive metabolomic characterization. This approach allowed for the integration of multiple plant matrices with distinct biochemical profiles into a single, reproducible formulation suitable for metabolomic evaluation.

### 2.3. Extraction of the Phytochemicals Present in BIOMEDER

For both UPLC–QTOF-MS and UHPLC–QTRAP-MS analysis, 50 mg of BIOMEDER (per triplicate) was weighed and extracted with 500 µL of MeOH:H_2_O (80:20) containing 0.1% formic acid (Merck, Darmstadt, Germany). The mixture was vortexed (Cole Parmer, USA) and sonicated in a bath (Merck, Germany) for three 30-s intervals, and then supernatants were filtered through 0.22 µm PVDF filters (Millipore, Darmstadt, Germany) before being analyzed.

### 2.4. UPLC–QTOF-MS Untargeted Analysis of Phytochemicals

Untargeted metabolomic profiling was conducted using a Waters ACQUITY UPLC I-Class System (Waters Corporation, Milford, MA, USA) coupled to a Bruker Daltonics maXis Impact quadrupole time-of-flight (QTOF) mass spectrometer (Bruker Daltonics GmbH, Bremen, Germany), operating at a resolution of ≥55,000 FWHM. Electrospray ionization (ESI) was applied in both positive [ESI (+)] and negative [ESI (−)] polarity modes. Chromatographic separation was achieved on a reversed phase column, Poroshell 120 Bonus—RP C18 column (3.0 × 100 mm, 2.7 µm; Agilent Technologies, Waldbronn, Germany) at a constant flow rate of 0.3 mL·/min. The mobile phase consisted of solvent A (H_2_O containing 0.1% formic acid, pH ≈ 3.2; PanReac AppliChem, Barcelona, Spain) and solvent B (acetonitrile with 0.1% formic acid; J.T. Baker, NJ, USA). The chromatographic gradient started with 1% B at 0 min, linearly increased to 18% at 10 min, linearly to 38% at 16 min, linearly to 95% at 22 min and finally returned to 1% B, which was held until the end of the run at 28 min. Nitrogen was used as both the desolvation and nebulization gas, with flow and pressure settings of 9 L·/min and 2.0 bar, respectively. The desolvation temperature was maintained at 200 °C, while the column compartment was held at 40 °C. The ESI source voltage was set to 4.5 kV for positive ionization mode. High-resolution QTOF-MS experiments were performed in broadband collision-induced dissociation mode (+bbCID), with the collision energy fixed at 24 eV under ESI (+). Data acquisition covered an *m*/*z* range of 50–1200 Da. Prior to each analytical batch, the instrument was externally calibrated using a 10 mM sodium formate solution delivered via a KNAUER Smartline Pump 100 (KNAUER, Berlin, Germany) equipped with a pressure sensor. The calibration mixture was freshly prepared by combining 0.5 mL formic acid and 1.0 mL of 1.0 M sodium hydroxide in an isopropanol/Milli-Q water solution (1:1, *v*/*v*). This configuration ensured accurate mass calibration and reproducible detection of molecular features across the full dynamic range of the analysis. Data acquisition and quantitative analysis were performed using Bruker Compass Data Analysis software (version 4.2 SR2; Bruker Daltonik GmbH, Bremen, Germany).

### 2.5. UHPLC–QTRAP-MS Targeted Analysis of Phytochemicals

Targeted quantification of selected phytochemicals was performed using an Agilent 1290 Infinity II ultra-high-performance liquid chromatography (UHPLC) system (Agilent Technologies, Waldbronn, Germany) coupled to a QTRAP 6500+ hybrid triple quadrupole/linear ion trap mass spectrometer (Sciex, Wilmington, DE, USA). Chromatographic separation was achieved on a reversed phase column, Poroshell 120 Bonus–RP C18 column (3.0 × 100 mm, 2.7 µm; Agilent Technologies, Waldbronn, Germany). Each sample (2 μL) was injected at a flow rate of 0.4 mL/min. The mobile phase consisted of solvent A (0.1% formic acid in LC–MS-grade water) and solvent B (acetonitrile). Gradient elution was performed following the same chromatographic program applied in the untargeted analysis. The mass spectrometer was operated with a Turbo Ion Spray source in polarity switching mode (ESI^+^/ESI^−^). Source parameters were as follows: curtain gas, 35 psi; collision-activated dissociation (CAD) gas, 9 psi; ion spray voltage, +5500 V/−4500 V; source temperature, 400 °C; gas 1, 60 psi; gas 2, 60 psi. The total run time was 14 min. Data acquisition was conducted in multiple reaction monitoring (MRM) mode using the Scheduled MRM™ algorithm with a 60 s detection window. For each analyte, two transitions were monitored: one for quantification (SRM 1) and one for confirmation (SRM2). Data acquisition and quantitative analysis were performed using Sciex OS software (version 2.2.0.5738; Sciex, Wilmington, DE, USA). The method exhibited limits of quantification (LOQ) ranging between 10 and 10,000 ng/L. Average recoveries were within the accepted range of 70–120%, with relative standard deviations (RSDs) ≤ 20%. Given the high diversity of the analyzed compounds and their distinct physicochemical characteristics, the recovery results were considered satisfactory. Intra-day repeatability (*n* = 3) confirmed that approximately 85% of the evaluated metabolites showed the precision acceptance criteria (RSD ≤ 20%), ensuring analytical reliability across the dataset.

### 2.6. ^1^H-NMR Analysis of Nutrients and Primary Metabolites

Targeted metabolomic profiling of BIOMEDER was performed following the procedure described by Van der Sar et al. (2013) [18], with minor adaptations. Triplicate samples were analyzed on a 500 MHz Bruker spectrometer (Bruker Biospin, Rheinstetten, Germany) equipped with a broadband 5 mm N_2_ CryoProbe Prodigy BBO. Measurements were conducted at 298 ± 0.1 K without sample spinning, using four dummy scans followed by 32 acquisition scans. The acquisition parameters were as follows: FID size, 64 K; spectral width, 12.4345 ppm; receiver gain, 28.5; acquisition time, 2.18 s; relaxation delay, 2 s; and line broadening, 0.50 Hz. Data collection employed the NOESY-presaturation pulse sequence (Bruker 1D, noesypr1d), in which water suppression was achieved by low-power irradiation at the water resonance during both relaxation and mixing periods. During data processing, multilevel deconvolution algorithms were used to minimize spectral noise, followed by baseline correction and area integration for signal quantification. These operations produced a representative ^1^H-NMR metabolic fingerprint, providing an overview of the most abundant metabolites, with chemical shifts (δ) expressed in parts per million (ppm). The NMR system generated frequency–intensity plots corresponding to the sample acquisition spectra. The resulting ^1^H-NMR spectra were analyzed using Chenomx NMR Suite (version 10.0; Chenomx Inc., Edmonton, AB, Canada) for metabolite identification and quantification. Deuterated trimethylsilylpropionic acid sodium salt (TSP-d_4_) served as the internal chemical shift reference, and sample pH was adjusted to approximately 6 prior to analysis. The software enabled accurate detection and quantification of metabolites at concentrations exceeding 10 μM.

### 2.7. SPME-GC–MS Analysis of VOCs Profile

Volatile metabolites from the plant-based BIOMEDER formulation were extracted and analyzed using solid-phase microextraction (SPME) combined with gas chromatography–mass spectrometry (GC–MS). The contents of one capsule (300 mg) were transferred into 20 mL headspace vials and loaded onto an autosampler (MPS Dual Head Robotic System, Gerstel, Mellinghofen, Germany). Each analysis was performed in triplicate to ensure reproducibility. GC–MS analyses were carried out on an HP 8890B gas chromatograph coupled to an HP 5977B quadrupole mass spectrometer (Agilent Technologies, Palo Alto, CA, USA). Prior to extraction, vials were equilibrated for 30 min at 25 °C. A DVB/Carboxen/PDMS (50/30 μm) SPME fiber (Supelco, Bellefonte, PA, USA) was preconditioned at 50 °C for 20 min and then exposed to the sample headspace for 10 min. Volatile desorption occurred in the GC injector at 250 °C for 3 min using a split ratio of 1:10 with an ultra-inert liner (Agilent Technologies). A VF-WAXms capillary column (30 m × 0.25 mm × 0.25 μm, Agilent Technologies) was employed. The oven temperature was programmed as follows: initial temperature 40 °C (5 min), increased at 4 °C·min^−1^ to 225 °C, and held for 51.25 min. The MS detector operated in scan mode (*m*/*z* 30–500, EI 70 eV) with a source temperature of 230 °C and a scan rate of 1.6 scans/s (cycle time: 622.9 ms). Raw data were processed using MassHunter Qualitative Analysis software (version B.10.00, Service Pack 1; Agilent Technologies). Compound detection combined the “molecular feature extraction” and “deconvolution” algorithms, selecting peaks with an absolute signal intensity ≥ 5000 counts from both GC and MS spectra. Tentative identification of volatiles was achieved by matching experimental spectra with the Wiley11N17 and NIST20 libraries (Wiley, Chichester, UK), accepting only those with a similarity index ≥ 80% and confirmed retention indices consistent with published data.

### 2.8. Data Treatment

The BIOMEDER extract was analyzed in triplicate for each metabolomics platform to ensure reproducibility. For every detected metabolite, standard deviations were calculated and are presented in the corresponding tables. As previously described by Vallejo et al. [19], processing of the VOCs dataset—including feature extraction, deconvolution, and alignment—was performed using Agilent Profinder software (version B.06.00; Agilent Technologies, Waldbronn, Germany). The resulting CEF files were subsequently imported into Mass Profiler Professional (MPP, revision B.14.09.01; Agilent Technologies, Santa Clara, CA, USA) for downstream statistical evaluation.

## 3. Results and Discussion

### 3.1. Phytochemical Profiling: Integration of High-Resolution and High-Sensitivity Platforms

A platform specially designed to provide maximum sensitivity (UHPLC-QTRAP) was used with pure standards. Meanwhile, an ID3 identification was established using another platform that provides maximum resolution (exact mass, isotopic distribution, and MS/MS) to identify unknown metabolites without standards (UPLC-QTOF). These platforms are ideal for integrating results as they are complementary. BIOMEDER (300 mg) obtained from orange, lemon, olive leaves, carob pods, shiitake mushroom, and salicornia by-products exhibited a complex and rich phytochemical profile. Combining data from high-resolution UPLC-QTOF and high-sensitivity UHPLC-QTRAP platforms enabled both qualitative and quantitative identification of phenolic compounds, confirming the presence of 37 metabolites (Table 1; Figure S1). Among the most abundant constituents were luteolin-7-O-glucoside (45.1 mg/300 mg), hesperidin (34.2 mg/300 mg), eriocitrin (24.2 mg/300 mg), oleuropein (10.8 mg/300 mg) and nobiletin (10.7 mg/300 mg). These levels are notably higher than typical concentrations reported in single-source citrus matrices [20,21,22]. Hesperidin, a major flavanone in citrus peels, is well-documented for its antioxidant and vasoprotective effects [23,24]. Eriocitrin, predominant in lemon tissues, demonstrated high radical scavenging capacity and is considered a marker compound for nutraceutical use [24,25]. The significant presence of luteolin-7-O-glucoside, a known anti-inflammatory flavone, further supports the potential bioactivity of the powder [26]. Notably, polymethoxylated flavones (PMFs) such as nobiletin, tangeretin, and sinensetin were detected in high concentrations. PMFs are primarily found in citrus peels and are associated with anti-obesity, neuroprotective, and anticancer activities [26]. Their lipophilic nature complements the water-soluble flavonoid glycosides in the formulation, potentially broadening bioactivity spectra [27]. Moderate amounts of quercetin, rutin, and apigenin were also detected, reflecting the contribution of citrus and carob matrices [25]. The presence of benzoic and p-coumaric acids suggests a secondary contribution from salicornia and shiitake, which are known to contain hydroxybenzoic and hydroxycinnamic acids [1,14]. Interestingly, compounds such as verbascoside (olive leaves) and poncirin (carob pods) were present at low or undetectable levels, likely due to the relative dilution in the final formulation or limitations in extraction efficiency. Nonetheless, the overall phytochemical fingerprint confirms that the powder retained and, in some cases, concentrated bioactive markers from all six input matrices. These results position BIOMEDER as a promising candidate for functional food and nutraceutical applications, offering a wide spectrum of bioactive flavonoids, phenolic acids, and PMFs with documented roles in oxidative stress reduction, inflammation modulation, and metabolic health [13,14,15]. Ongoing studies will assess BIOMEDER’s bioaccessibility, bioavailability, and potential increasing interactions among its phytochemical constituents.

The quantitative evaluation of the phenolic compounds reveals both similarities and deviations from previously reported concentrations in single-source byproducts. Among flavanone glycosides, hesperidin (34.2 mg/300 mg; ~113 mg/g) was particularly abundant, exceeding typical values reported for orange peel extracts (20–50 mg/g) [21,28]. Likewise, eriocitrin (24.2 mg/300 mg; ~80 mg/g) was higher than the range usually found in lemon peel residues (15–40 mg/g) [24,25], while neoeriocitrin (0.5 mg/300 mg; ~1.7 mg/g) and diosmin (1.0 mg/300 mg; ~3.3 mg/g) fell within previously described ranges for lemon tissues [27,29]. Naringin (3.3 mg/300 mg; ~11 mg/g) and isosakuranetin (3.0 mg/300 mg; ~10 mg/g) were present at levels comparable to reported averages in citrus peels [30]. Flavone glycosides also contributed significantly: luteolin-7-O-glucoside (45.1 mg/300 mg; ~150 mg/g) was strikingly higher than the 5–15 mg/g usually reported for citrus matrices [30], while vitexin (3.0 mg/300 mg; ~10 mg/g), orientin (3.5 mg/300 mg; ~12 mg/g), rhoifolin (2.8 mg/300 mg; ~9 mg/g), and scoparin (1.1 mg/300 mg; ~4 mg/g) matched concentrations detected in diverse citrus germplasm collections [30,31]. Apigenin (1.1 mg/300 mg; ~4 mg/g) also aligned with literature values in orange and lemon byproducts [10]. BIOMEDER was especially rich in polymethoxylated flavones (PMFs). Nobiletin (10.7 mg/300 mg; ~36 mg/g) and tangeretin (3.5 mg/300 mg; ~12 mg/g) yielded combined values (~48 mg/g) above those typically found in orange peel powders (10–30 mg/g) [27,31]. Additionally, sinensetin (6.8 mg/300 mg; ~23 mg/g), pentamethoxyflavone (11.1 mg/300 mg; ~37 mg/g), tetramethoxyflavone (11.9 mg/300 mg; ~40 mg/g), and heptamethoxyflavone (6.7 mg/300 mg; ~22 mg/g) expanded the PMF profile beyond commonly reported values for single citrus extracts [27]. Among flavonols, rutin (6.5 mg/300 mg; ~22 mg/g) and quercetin (3.3 mg/300 mg; ~11 mg/g) were within the range described for citrus peels and seeds (5–20 mg/g) [32,33], while isorhamnetin (0.1 mg/300 mg; ~0.3 mg/g) appeared in trace levels, similar to reports in salicornia and citrus tissues [18,19]. The secoiridoid oleuropein (10.8 mg/300 mg; ~36 mg/g) was present at higher levels than those previously described in olive leaf extracts (8–15 mg/g) [32,34], confirming the olive contribution. Verbascoside, although present in low concentration (0.1 mg/300 mg; ~0.3 mg/g), is also characteristic of olive byproducts [33]. In terms of phenolic acids, gallic acid (3.0 mg/300 mg; ~10 mg/g), p-coumaric acid (3.2 mg/300 mg; ~11 mg/g), and benzoic acid (3.2 mg/300 mg; ~11 mg/g) were moderately abundant, consistent with carob, shiitake, and salicornia sources [35,36,37,38]. Minor levels of caffeic acid (0.5 mg/300 mg; ~1.7 mg/g), vanillic acid (0.5 mg/300 mg; ~1.7 mg/g), and ellagic acid (0.3 mg/300 mg; ~1.0 mg/g) also align with mushroom and halophyte matrices [37,39,40]. Regarding limonoids, limonin (2.9 mg/300 mg; ~10 mg/g) and nomilin (0.5 mg/300 mg; ~1.7 mg/g) were detected at concentrations compatible with citrus seed and peel fractions [21,25]. Overall, these comparisons indicate that BIOMEDER not only preserved the typical phytochemical fingerprints of citrus byproducts but also achieved higher concentration of several key compounds (hesperidin, eriocitrin, luteolin-7-O-glucoside, oleuropein and PMFs) beyond levels usually reported in single-source extracts. This higher concentration might reflect accumulation from the combination of diverse plant residues.

### 3.2. Metabolomics Multiplatform: ^1^H-NMR Analysis of Nutrients and Primary Metabolites

The targeted ^1^H-NMR profiling of BIOMEDER revealed a complex matrix of sugars, organic acids, amino acids, and osmoprotectants (Table 2; Figure S2). The major soluble metabolites identified were consistent with the metabolic spectral fingerprints reported for the individual source materials. Sugars such as fructose (761 mg/g), glucose (570.7 mg/g), and sucrose (927.2 mg/g) were the main metabolites found, reflecting contributions from citrus and carob residues. These simple carbohydrates are not only nutritional components but also exhibit prebiotic effects, supporting beneficial gut microbiota [41]. Trehalose (164.3 mg/g), identified in both carob and shiitake, and mannitol (148.7 mg/g), a sugar alcohol found in shiitake and olive leaves by-products, are known for their antioxidant and cytoprotective properties in functional food applications [42,43,44]. Among organic acids, citrate (189.0 mg/g), malate (86.9 mg/g), and quinic acid (202.9 mg/g) were the most abundant. These compounds play central roles in energy metabolism and contribute to flavor, metal chelation, and physiological effects such as nephroprotection [45,46,47]. The presence of quinic acid supports the contribution of olive and carob matrices and reinforces BIOMEDER potential as a source of shikimate-derived metabolites. Osmoprotectants and metabolic regulators were also detected at relevant concentrations. Myo-inositol (220.5 mg/g), betaine (53.4 mg/g), and proline (44.2 mg/g) are known to play key roles in cellular stress response and osmoregulation—especially relevant in halophytes like salicornia [48]. Choline (10.0 mg/g), another osmolyte, contributes to methyl group metabolism and neuroprotection. Amino acids such as arginine (33.4 mg/g), asparagine (28.5 mg/g), glutamate (28.2 mg/g), and branched-chain amino acids (leucine, isoleucine, valine) were present at moderate levels. These compounds support a variety of physiological functions, including nitric oxide production (arginine), neurotransmitter synthesis (glutamate), and muscle metabolism (BCAAs) [48,49,50,51]. The detection of γ-aminobutyric acid (GABA, 2.5 mg/g), though low in concentration, is noteworthy given its role in modulating blood pressure and stress responses [52]. Overall, the ^1^H-NMR results confirm that BIOMEDER contains a metabolite pool with both nutritional and functional relevance. The metabolite classes detected are consistent with previous reports on the individual by-products, and their simultaneous presence supports the hypothesis that combining diverse plant sources enhances compositional diversity and functional potential.

### 3.3. Metabolomics Multiplatform: SPME-GC–MS Analysis of VOCs Profile

The volatile fraction of the BIOMEDER was analyzed via SPME-GC-MS per triplicate, identifying 38 VOCs (score > 80%) and revealing a diverse profile dominated by terpenes, aldehydes, acids, and alcohols—compounds with both aromatic and bioactive potential. Monoterpenes such as D-limonene, γ-terpinene, linalool, and α-terpineol were the predominant volatiles, especially in the citrus-derived fractions (Table 3; Figure S3). D-limonene, the most abundant compound, is widely recognized for its citrus aroma and has been associated with anti-inflammatory and chemopreventive activities [53,54]. Linalool and α-terpineol contribute floral and herbal notes and are known for antimicrobial and antioxidant effects, supporting their use in functional food formulations [54]. Aldehydes such as hexanal and nonanal, likely originating from olive leaves by-products, impart green and fruity notes and are commonly used as markers of freshness and lipid oxidation status in olive matrices [55]. Carob contributed furans and sugar degradation products responsible for roasted and sweet aromatic notes, consistent with their sensory profile [56]. Salicornia fractions were characterized by short-chain organic acids (e.g., acetic acid, 2-methyl-propanoic acid), which are typical of halophytes and contribute fermentation-like or marine-associated aromas. These findings are in line with recent reports on the volatile composition of halophyte species [57]. Although VOCs from shiitake were less abundant, C8 compounds such as 1-octen-3-ol were detected and represent typical “mushroom-like” volatiles. These compounds enhance umami perception and have been studied for their bioactivity in immune regulation and oxidative defense [58]. Overall, the VOC profile demonstrates that BIOMEDER preserves aromatic complexity and bioactive volatiles from all components. This diversity not only contributes to sensory appeal but may also enhance BIOMEDER antioxidant, antimicrobial, and anti-inflammatory properties, making it suitable for functional food and nutraceutical applications.

### 3.4. Integrative Discussion: Functional Potential and Scientific Relevance of BIOMEDER

The results obtained across metabolomics platforms demonstrate that combining Mediterranean plant-based by-products into a single formulation significantly enhances the diversity and abundance of bioactive compounds. Each matrix contributed a unique and complementary profile: citrus residues enriched with flavanones and polymethoxylated flavones; olive leaves provided hydroxytyrosol derivatives; carob pods contributed polyphenols and sugars; shiitake mushrooms added amino acids and fungal-specific volatiles; and salicornia offered osmoprotectants and halophyte-derived metabolites. The combing use of an untargeted (UPLC-QTOF-MS) and targeted (UHPLC-QTRAP-MS. ^1^H-NMR. SPME-GC-MS) metabolomics approach enabled comprehensive characterization of phytochemicals, nutrients, and volatiles—critical for understanding the potential bioactivity and safety of the formulation.

The compositional profile of BIOMEDER reveals a formulation with functional potential arising from the combination of six agro-industrial by-products. The high content of flavanones, flavones, flavonols, and secoiridoids, together with amino acids, organic acids, and volatiles, might define BIOMEDER as a functional bioactive matrix capable of modulating oxidative stress, inflammation, metabolic balance, and gut–brain axis signaling [58,59]. Antioxidant and Redox-Modulating Effects: The abundant luteolin-7-O-glucoside, eriocitrin, and hesperidin confer potent antioxidant activity, acting through both radical scavenging and enhancement of endogenous defense systems via activation of the Nrf2–ARE pathway [60]. Oleuropein further contributes by modulating redox-sensitive signaling and preventing lipid oxidation, while polymethoxylated flavones (nobiletin, tangeretin) inhibit oxidative enzyme systems such as NADPH oxidase, thereby stabilizing cellular redox balance [61]. The presence of complementary antioxidants across different polarity ranges supports the observed strong antioxidant potential of BIOMEDER. Anti-Inflammatory and Endothelial-Protective Activity: The polyphenol-rich composition of BIOMEDER also underpins its anti-inflammatory and endothelial-protective capacity. Luteolin-7-O-glucoside and hesperidin inhibit NF-κB and COX-2 expression, reducing cytokine release (IL-6, TNF-α), while eriocitrin reinforces these actions through iNOS inhibition and enhancement of nitric oxide bioavailability [62,63]. The inclusion of oleuropein, with its well-documented cardioprotective and anti-inflammatory activity, complements the citrus-derived effects, mirroring the vascular benefits associated with the Mediterranean diet [64]. Metabolic Modulation: Several of BIOMEDER’s principal metabolites are involved in lipid and glucose homeostasis. Hesperidin and nobiletin activate AMPK and PPARα signaling, enhancing β-oxidation and reducing hepatic lipid accumulation [65]. Eriocitrin improves insulin sensitivity through GLUT4 activation and suppression of gluconeogenesis [66], while oleuropein modulates cholesterol metabolism and bile acid synthesis [67]. Together, these mechanisms suggest that BIOMEDER could support cardiometabolic health through multi-target metabolic regulation. Neuroprotective and Gut Microbiota Effects: Recent studies have shown that luteolin derivatives exert neuroprotective effects by attenuating oxidative stress and inflammation via MAPK and PI3K/Akt pathways [68]. Moreover, hesperidin and oleuropein modulate gut microbiota composition by promoting beneficial Lactobacillus and Bifidobacterium species and reducing proinflammatory Enterobacteriaceae [69,70]. This bidirectional interaction between gut and brain function might suggest that BIOMEDER could contribute to neuroprotective and microbiota-balanced functional foods. Functional Synergy and Application Potential: The coexistence of hydrophilic flavonoids (eriocitrin, hesperidin) with lipophilic PMFs (nobiletin, tangeretin) enhances intestinal absorption and bioavailability, while amino acids (GABA, proline, arginine) and organic acids (citric, malic) stabilize polyphenols and modulate vascular tone [71]. These synergistic interactions might create a multi-layered bioactive system with complementary mechanisms across oxidative, inflammatory, and metabolic pathways. In summary, BIOMEDER might represent a polyphenol-rich, functional ingredient with potential applications in functional foods and nutraceuticals targeting oxidative stress, chronic inflammation, metabolic disorders, and gut–brain axis balance.

Nevertheless, the results must be interpreted within the scope of analytical profiling. While compositional data suggest health-promoting potential, biological validation remains essential. Our advancing studies will evaluate BIOMEDER’s bioactivity in vivo, focusing on anti-inflammatory, metabolic, and microbiota-related effects. In addition, studies on safety assessments such as chemical contaminants are critical for regulatory approval and product development. In summary, this work demonstrates that the integration of diverse Mediterranean by-products, coupled with multiplatform metabolomics, yields a promising supplement in terms of compositional richness, functional potential, and sustainability. The methodology and insights generated provide a wide base for the balanced design of new ingredients derived from plant-based residues which might be multifunctional.

### 3.5. Comparative Discussion of Analytical Platforms

The present study employed four advanced analytical platforms—HR-UHPLC-QTOF-MS, HS-UHPLC-QTRAP-MS, ^1^H-NMR spectroscopy and SPME-GC-MS—to comprehensively characterize the bioactive profile of plant-derived by-products. These matrices, rich in diverse phytochemicals such as polyphenols, flavonoids, volatile compounds, amino acids, and organic acids, require a multimodal analytical strategy to capture their chemical complexity. Thus, the combination of HR-UHPLC-QTOF-MS and HS-UHPLC-QTRAP-MS platforms was most effective for polyphenols, allowing for high-coverage and high-precision identification of major flavonoids and phenolic acids. In contrast, ^1^H-NMR offered robust quantification for amino acids and organic acids, with minimal sample preparation. However, its lower sensitivity compared to MS-based techniques limits detection of low-abundance metabolites. For the volatile fraction, SPME-GC-MS emerged as an ideal tool due to its minimal sample handling and high sensitivity to aroma-active compounds. Nevertheless, it is dependent on fiber coating selectivity and can exhibit biases. GC-MS, when used independently, allowed for complementary profiling of thermally stable small molecules, but it lacks the capacity for larger, non-volatile compounds relevant to functional food analysis. Importantly, the complementarity of these techniques allowed for cross-validation of metabolite identities, expanded coverage, and a more holistic interpretation of the nutritional and functional properties of the studied matrices. This multi-platform approach enhances analytical reliability and helps overcome the inherent trade-offs of individual methods.

## 4. Conclusions

In conclusion, a metabolomics multiplatform approach was essential to comprehensively identify and quantify the diverse phytochemicals, nutrients, and volatile compounds present in the newly developed lyophilized powder from Mediterranean plant-based by-products. The combination of citrus, olive leaves, carob pods, mushroom, and salicornia residues proved to be an alternative strategy to broaden the spectrum of bioactive compounds, supporting the hypothesis as a source of health-promoting effects and reinforcing their potential use in food and nutraceutical applications. This study highlights the value of integrating diverse agro-industrial by-products into formulations that potentially might be multifunctional which align with circular economy principles. However, further studies are required to validate the biological relevance of the observed phytochemical and nutritional diversity to ensure its translation from lab-scale development to practical food or supplement applications.

## Figures and Tables

**Table 1:** List of 37 metabolites identified in BIOMEDER combining two analytical platforms: (i) HR high resolution (UPLC-QTOF-MS) and (ii) HS high sensitivity (UHPLC-QTRAP-MS).

Number	Metabolites	RT	Polarity	Formula	Experimental *m*/*z*	MS/MS Fragment	Concentration (mg/capsule d.w. ± s.d.)	Concentration (mg/g d.w. ± s.d.)	%	Platform
1	Gallic acid *	6.7	Negative	C_7_H_6_O_5_	169.0	124.9	3.0 ± 0.4	9.9 ± 1.4	1.5	HS
2	Gallocatechin *	9.1	Positive	C_15_H_14_O_7_	307.1	139.0	0.4 ± 0.1	1.4 ± 0.3	0.3	HS
3	Catechin *	11.6	Positive	C_15_H_14_O_6_	291.0	139.0	0.8 ± 0.1	2.6 ± 0.3	0.6	HS
4	p-coumaric acid *	11.8	Negative	C_9_H_8_O_3_	163.0	118.9	3.2 ± 0.3	10.6 ± 0.9	1.1	HS
5	Vicenin-2	12.1	Positive	C_27_H_30_O_15_	595.1685	†	-	-	1.3	HR
6	Vanillic acid *	12.2	Negative	C_8_H_8_O_4_	167.1	151.9	0.5 ± 0.1	1.7 ± 0.3	0.2	HS
7	Stellarin-2	12.5	Positive	C_28_H_32_O_16_	625.1784	505.1354	-	-	0.6	HR
8	Caffeic acid *	13.1	Negative	C_9_H_8_O_4_	179.0	135.0	0.5 ± 0.1	1.7 ± 0.3	0.3	HS
9	Rutin *	13.7	Negative	C_27_H_30_O_16_	609.1	301.0	6.5 ± 0.5	21.5 ± 1.7	3.3	HS
10	Eriocitrin *	13.8	Negative	C_27_H_32_O_15_	595.1	286.9	24.2 ± 1.1	79.9 ± 3.6	12.6	HS
11	Verbascoside	14.0	Positive	C_29_H_36_O_15_	625.2122	†	-	-	0.1	HR
12	Orientin	14.1	Positive	C_21_H_20_O_11_	449.1094	413.1847	-	-	2.6	HR
13	Luteolin-7-O-glucoside *	14.2	Negative	C_21_H_20_O_11_	447.0	285.0	45.1 ± 3.1	148.8 ± 10.2	21.7	HS
14	Neoeriocitrin *	14.1	Negative	C_27_H_32_O_15_	595.1	286.9	0.5 ± 0.1	1.7 ± 0.3	0.1	HS
15	Scoparin	14.3	Positive	C_22_H_22_O_11_	463.1239	†	-	-	0.8	HR
16	Rhoifolin	14.5	Positive	C_27_H_30_O_14_	579.1716	379.0961	-	-	1.2	HR
17	Naringin	14.6	Positive	C_27_H_32_O_14_	581.1872	273.0759	-	-	1.4	HR
18	Diosmin	14.9	Positive	C_28_H_32_O_15_	609.1823	301.0707	-	-	0.8	HR
19	Hesperidin *	15.0	Negative	C_28_H_34_O_15_	609.2	301.0	34.2 ± 2.8	112.9 ± 9.2	15.7	HS
20	Vitexin	15.1	Positive	C_21_H_20_O_10_	433.1134	†	-	-	1.5	HR
21	Benzoic acid *	15.2	Negative	C_7_H_6_O_2_	286.9	76.9	3.2 ± 0.2	10.6 ± 0.9	2.0	HS
22	Ellagic acid *	15.4	Negative	C_14_H_6_O_8_	301.2	228.9	0.3 ± 0.1	0.9 ± 0.3	0.1	HS
23	Nomilin	15.6	Positive	C_28_H_34_O_9_	515.2283	†	-	-	0.3	HR
24	Oleuropein	16.1	Positive	C_25_H_32_O_13_	541.1931	379.1389	-	-	4.9	HR
25	Poncirin	16.9	Positive	C_28_H_34_O_14_	595.2031	†	-	-	0.9	HR
26	Quercetin *	17.7	Negative	C_15_H_10_O_7_	301.0	150.9	3.3 ± 0.3	10.9 ± 0.9	0.4	HS
27	Sinensetin *	18.2	Negative	C_20_H_20_O_7_	373.1	312.0	6.8 ± 0.4	22.4 ± 1.3	3.5	HS
28	Naringenin *	18.5	Positive	C_15_H_12_O_5_	273.0	153.0	0.3 ± 0.1	0.9 ± 0.3	0.1	HS
29	Nobiletin *	19.3	Positive	C_21_H_22_O_8_	403.0	373.0	10.7 ± 1.1	35.3 ± 3.3	4.8	HS
30	3,5,7,3′,4′-Pentamethoxyflavone	19.5	Positive	C_20_H_20_O_7_	373.1309	329.1019	-	-	2.0	HR
31	Limonin	19.7	Positive	C_26_H_30_O_8_	471.2021	425.1960	-	-	1.7	HR
32	Isorhamnetin *	19.9	Negative	C_16_H_12_O_7_	315.0	299.9	0.1 ± 0.0	0.3 ± 0.0	0.1	HS
33	Apigenin *	20.1	Positive	C_15_H_10_O_5_	271.0	153.0	1.1 ± 0.1	3.3 ± 0.3	0.5	HS
34	6,7,8,4′-Tetramethoxyflavone	20.2	Positive	C_19_H_18_O_6_	343.1196	†	-	-	5.2	HR
35	3,5,6,7,8,3′,4′-Heptamethoxyflavone	20.4	Positive	C_22_H_24_O_9_	433.1510	418.1260	-	-	3.2	HR
36	Tangeretin *	20.7	Positive	C_20_H_20_O_7_	373.1	343.1	3.5 ± 0.2	11.6 ± 0.7	2.2	HS
37	Isosakuranetin *	21.5	Positive	C_16_H_14_O_5_	287.0	153.1	3.0 ± 0.3	9.9 ± 0.9	0.4	HS

* Confirmed metabolites with pure standards were quantified by HS platform. † Metabolites without fragments were identified with level 3 according to the metabolomics consortium [19] and with a mSigma value < 50. Metabolites with fragments were tentatively confirmed by exact mass, isotopic pattern, fragments (+bbCID at 24 ev of collision energy), libraries (Metlin, HMDB and CEU Mass Mediator) and bibliography. Samples were analyzed in triplicate ± standard deviation.

**Table 2:** ^1^H-NMR-detected metabolites and concentrations in BIOMEDER (mg/g d.w.).

Metabolites	Concentration (mg/g d.w. ± s.d.) *
4-Aminobutyrate	2.5 ± 0.9
Acetate	5.8 ± 0.5
Alanine	14.9 ± 0.7
Arginine	33.5 ± 12.9
Ascorbate	12.7 ± 0.1
Asparagine	28.5 ± 0.5
Aspartate	28.8 ± 3.5
Betaine	53.4 ± 1.9
Choline	10.2 ± 0.2
Citrate	189.1 ± 29.0
Formate	1.1 ± 0.0
Fructose	761.0 ± 23.5
Fumarate	2.8 ± 0.7
Glucose	570.7 ± 57.3
Glutamate	28.3 ± 1.9
Glutamine	21.0 ± 1.0
Isoleucine	6.3 ± 0.7
Lactate	6.5 ± 0.9
Leucine	6.3 ± 0.7
Malate	86.9 ± 14.3
Mannitol	148.7 ± 6.3
Methylguanidine	1.8 ± 3.5
Myo-Inositol	220.5 ± 38.7
Ornithine	19.0 ± 1.2
Phenylalanine	3.9 ± 0.6
Proline	44.2 ± 0.3
Quinic acid	202.9 ± 31.9
Quinone	10.4 ± 3.3
Sucrose	927.2 ± 415.1
Trehalose	164.3 ± 26.4
Tryptophan	4.9 ± 0.7
Tyrosine	4.4 ± 0.5
Valine	13.7 ± 3.4

* Samples were analyzed in triplicate ± standard deviation.

**Table 3:** Main identified VOCs present in BIOMEDER.

Compound	Formula	Retention Time (Min)	Source	Bioactivity/Sensory Role	References
D-Limonene	C_10_H_16_	12.2	Citrus peel dominant monoterpene	Antioxidant, antimicrobial/aroma contributor	[53,56]
Linalool	C_10_H_18_O	24.3	Citrus, olive oil, floral aromas	Antioxidant, antimicrobial/sedative properties	[54]
Acetic acid	C_2_H_4_O_2_	21.9	Halophytes (Salicornia)	Contributes to acidity/aroma intensity	[57]
Propanoic acid. 2-methyl-	C_4_H_8_O_2_	25.4	Halophytes (Salicornia)	Volatile acid with pungent note	[57]
Terpinen-4-ol	C_10_H_18_O	25.9	Citrus	Antimicrobial/aroma contributor	[56]
5-Hepten-2-one. 6-methyl-	C_8_H_14_O	17.4	Citrus peel oils	Fresh fruity aroma	[53]
Nonanal	C_9_H_18_O	19.3	Olive oil, citrus	Fatty, floral aroma	[55]
Hexanal	C_6_H_12_O	8.1	Olive oil	Green/fresh aroma marker	[55]

Samples were analyzed per triplicate.

## Data Availability

The data are contained within the article.

## Supplementary Materials

The following supporting information can be downloaded at: https://www.mdpi.com/article/10.3390/foods15030565/s1, Figure S1: Extracted ion chromatogram of main bioactive metabolites identified in BIOMEDER by HR high resolution (UPLC-QTOF-MS) platform; Figure S2: 1H-NMR spectra of nutrients and primary metabolites identified in BIOMEDER; Figure S3: Total ion chromatogram of main VOCs identified in BIOMEDER by SPME-GC-MS platform.

## Abbreviations

The following abbreviations are used in this manuscript:

ACN	acetonitrile
BIOMEDER	BIOactive MEditerranean powDER
DMSO	dimethyl sulfoxide
DVB/CAR/PDMS	Divinylbenzene/Carboxen/Polydimethylsiloxane
ESI	Electrospray ionization
MeOH	methanol
NMR	Nuclear Magnetic Resonance
QTOF	Quadrupole Time of Flight
SPME	Solid-phase microextraction
VOCs	volatile organic compounds
