# Supplementary material for: Multiplatform Metabolomics for the Design and Characterization of a Mediterranean Plant-Based Lyophilized Powder from Agro-Industrial By-Products
Source: Foods. 2026 Feb 5;15(3):565. doi: 10.3390/foods15030565 (PMC12896481; doi:10.3390/foods15030565)

Figure S1. Extracted ion chromatogram of main bioactive metabolites identified in BIOMEDER by HR high resolution (UPLC-QTOF-MS) platform.

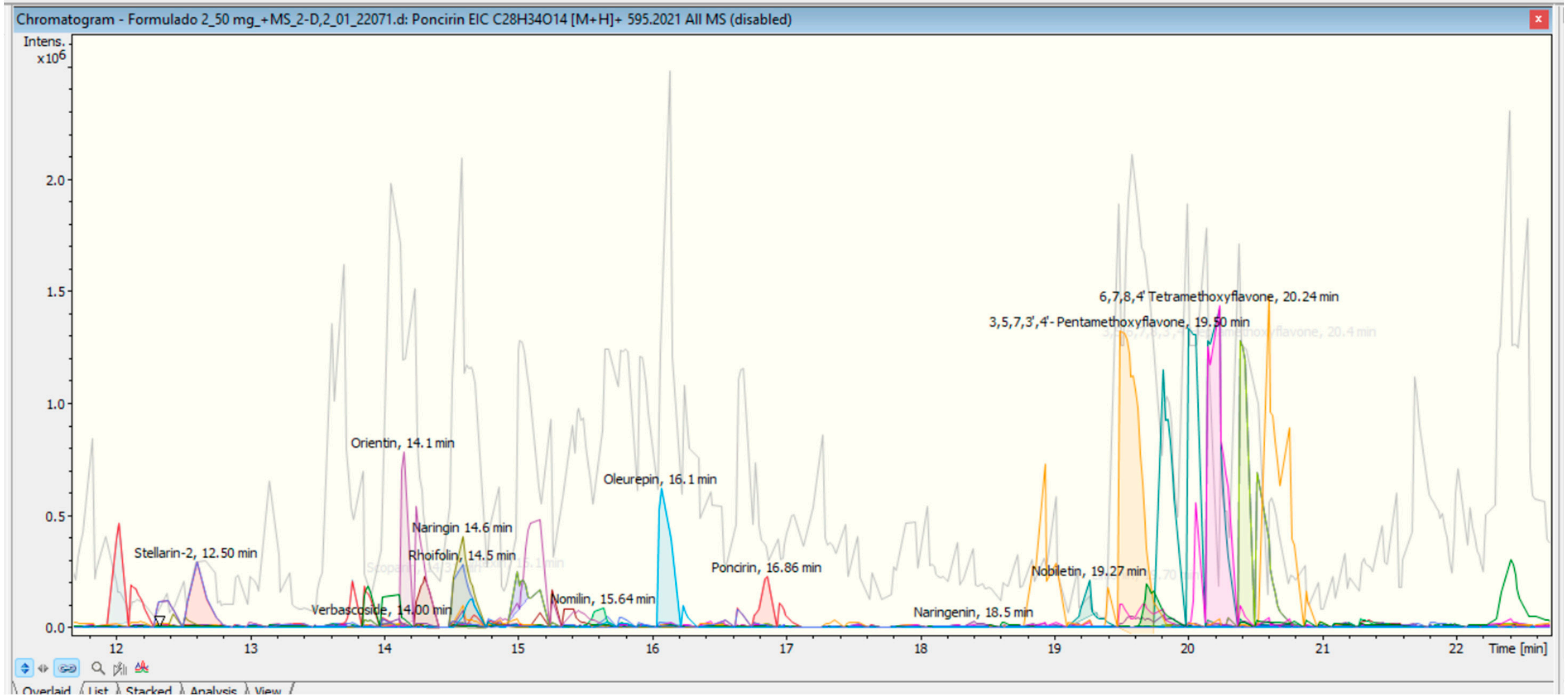

Figure S2. ^{1}H-NMR spectra of nutrients and primary metabolites identified in BIOMEDER.

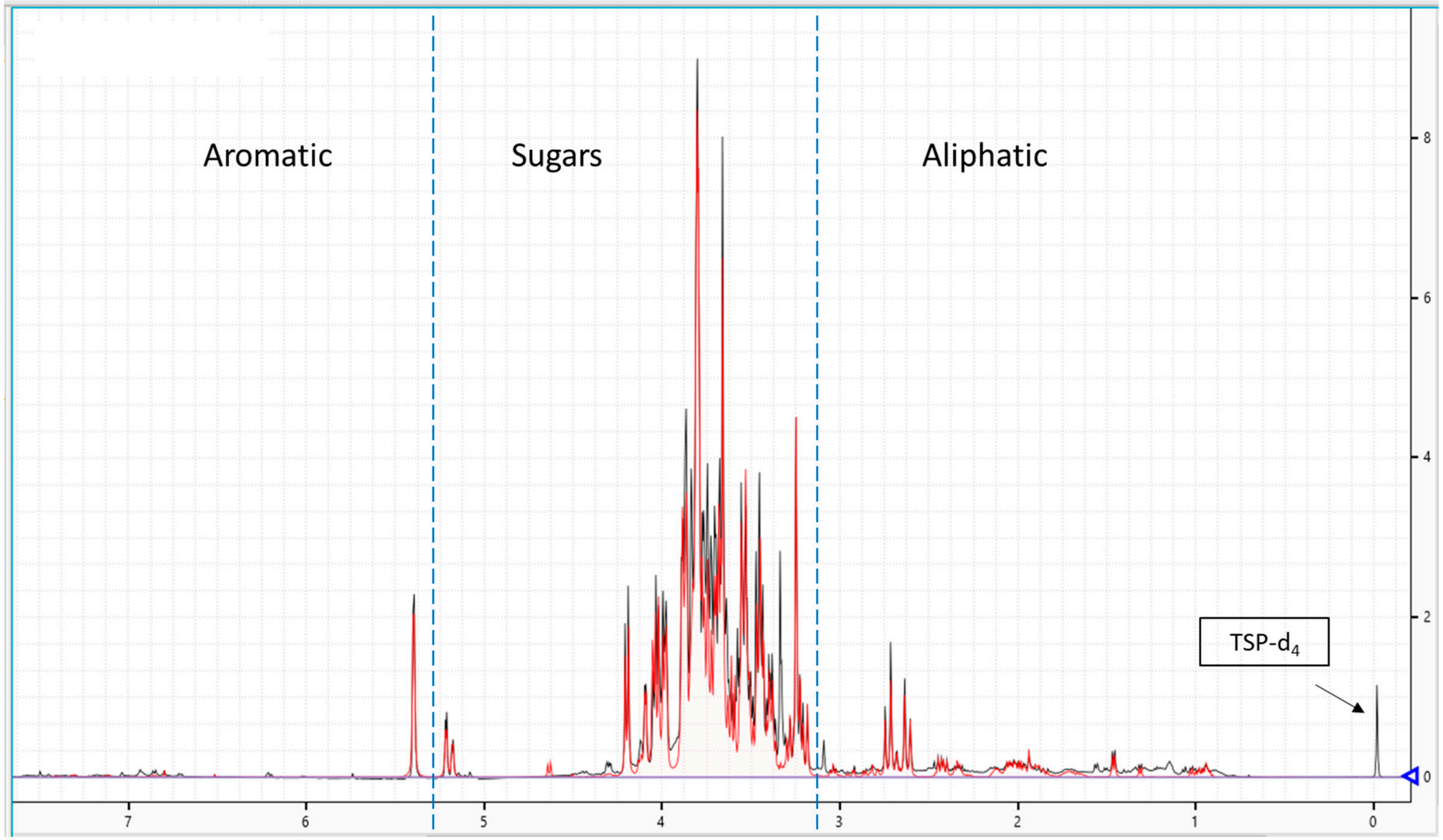

Figure S3. Total ion chromatogram of main VOCs identified in BIOMEDER by SPME-GC-MS platform.

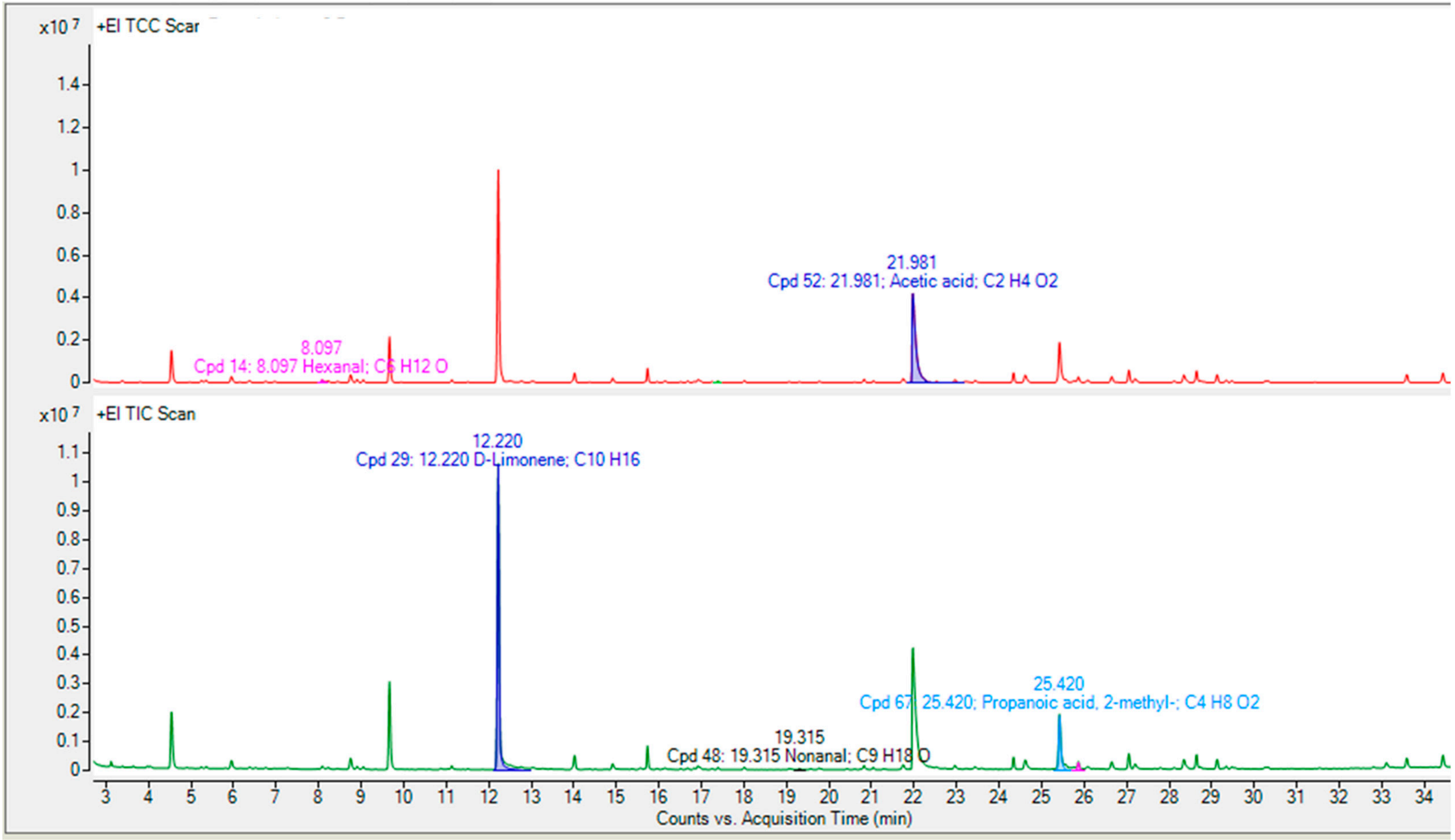

Supplement: Supplementary file 1 [file foods-15-00565-s001.zip › foods-4114815-supplementary.pdf]
